# Editorial: Repurposing for small population needs

**DOI:** 10.3389/fmed.2026.1836851

**Published:** 2026-05-20

**Authors:** Viviana Giannuzzi, Christine Fetro, Cécile Ollivier, Oscar Della Pasqua

**Affiliations:** 1 Fondazione per la Ricerca Farmacologica Gianni Benzi Onlus, Research and Innovation Department, Valenzano, Italy; 2 Foundation for Rare Diseases, Paris, France; 3 Critical Path Institute, Tucson, AZ, United States; 4 Clinical Pharmacology and Therapeutics Group, University College London, London, United Kingdom

**Keywords:** orphan medicines, pediatric medicines, rare diseases, repurposing, therapeutic needs

Despite rapid scientific and technological advancements, small populations, such as pediatric patients and those with rare diseases, still face substantial unmet needs. The Research Topic “Repurposing for small population needs” has collected 10 manuscripts, including original research, perspectives, mini reviews, and opinion articles. Collectively, they highlight how drug *repurposing* represents a therapeutic strategy for these populations, generating evidence while potentially making drug research and development faster, more cost-effective, and more efficient (1). This approach is based on the assumption that diseases share common biological targets and/or pathways and that every molecule, even the most optimized ones, is not entirely specific (pleiotropic drugs) (Paquot et al.). In fact, interest in, and the use of, drug repurposing reached unprecedented levels during the COVID-19 pandemic (2), even though opportunities have been missed to ensure accurate translation of findings into successful therapeutic interventions.

What first emerges from the manuscripts is that a clear definition of “drug repurposing” does not exist, nor is it formally established by law in the EU (Wang and Giannuzzi), despite regulatory initiatives by the European Commission's Expert Group on Safe and Timely Access to Medicines for Patients (STAMP) and the European Medicines Agency and the Heads of Medicines Agencies (HMA) pilot project (3). We are used to hearing synonyms such as “repurposing,” “reprofiling,” “repositioning,” “secondary indication,” and “reformulation”; all these terms focus on identifying “new uses” for a product outside the scope of its original medical indication, but this concept of “new use” is not well-defined. It could refer to a completely different disease in another therapeutic area, a different disease within the same therapeutic area, a new route of administration (for the same or a different disease), a new treatment regimen, or a new pharmaceutical form. It might also involve new patient populations, dosage forms, or routes of administration. The drug candidate for repurposing could be a medicine already marketed, including old medicines with expired intellectual property (IP) protection, a newly approved medicine, or a drug still under development that is found to be effective in the treatment of another condition. Wang and Giannuzzi propose qualifying a change in formulation as “reformulation” within the repurposing concept, even for a disease or condition already authorized in adults or children. Notably, a clear definition still appears to be lacking in the upcoming EU pharmaceutical legislation, despite the fact that it includes, for the first time, EU-wide rules on repurposing (4). This represents a major step forward in the pharmaceutical field, as data on off-label use are expected to be included in marketing authorization documents.

## Industrial and academic approaches

Both for-profit and not-for-profit organizations (academic institutions, patient associations, and not-for-profit research) are involved in repurposing research and development programmes to gather and generate evidence for new indications of medicines for small populations. This is also reflected in the Research Topic.

The development of medicinal products is becoming increasingly expensive, complex, and associated with a significant attrition rate. From a ***commercial*** perspective, drug repositioning is therefore appealing: pharmaceutical companies integrate it as a strategy in the life cycle management of their products, often referring to such activities as “secondary indications”. By leveraging a product's existing knowledge, the use of well-known drugs is expected to reduce time, costs, and development risks compared to *de novo* drug development (Jonker et al., Wang and Giannuzzi).

In the context of an ***academic*** research programme, a case study on the repurposing of the old drug guanabenz was conducted. Guanabenz, an old alpha-2 adrenergic receptor agonist used as an antihypertensive, was explored as a therapeutic strategy for amyotrophic lateral sclerosis, a rare and severe neurological disease (Ambrosini et al.).

However, while academic research often supports repurposing, the pharmaceutical industry tends to view it as a suboptimal investment, mostly because payers at the national level are reluctant to assign favorable prices that adequately reward the research efforts, even when these medicines prove effective (Paquot et al.). This is consistent with the findings of a systematic analysis of EU investments, such as the FP7 funding program, to date (Ruggieri et al.): 20 EU-funded projects based on public–private partnerships studying 24 active substances developed 22 repurposing plans to address unmet medical needs in the pediatric population. Of these, one pediatric investigation plan was officially discontinued due to commercial reasons, but financial difficulties/budgeting negatively impacted the conduct of eight PIPs, as reported by the project coordinators.

What is already known is that collaborations between academia and industry are usually established to carry out R&D processes for medicines with limited commercial interest. The Research Topic provides examples of drug repurposing achieved through effective ***collaborations between academia and industry, including public–private partnerships*** (Cortial et al., Jonker et al., Owen et al., Paquot et al., Ruggieri et al.). They aimed to undertake AI-driven initiatives (Cortial et al.), studying and developing medicines for children and rare disease patients (Jonker et al., Ruggieri et al.), creating tools and guides useful for repurposing (Owen et al.) (5), and identifying new therapeutic opportunities for ultra-rare patients (Paquot et al.).

## Scientific evaluations and regulatory decision-making

A repurposing R&D plan seeks novel therapeutic indications for known, authorized, or investigational drugs, but regulatory approval is needed to demonstrate efficacy, safety, and quality (with some data already available) and to authorize new indications with appropriate dosing, efficacy, and safety for specific populations. If needed, in the pediatric setting, it might also be relevant to develop new formulations that enable medicine use in target subsets, or new strengths to allow appropriate dosing. A recent example comes from the pediatric oncologic field, where a drug may be effective against multiple tumors due to its mechanism of action and/or its target.

However, repurposing often results in off-label use of medicines, providing clinicians with a unique therapeutic opportunity to treat patients when no alternative treatment is available, while only a limited portion of these uses ultimately becomes authorized.

In pediatric indications, a specific marketing authorization is available in the EU, namely, the pediatric-use marketing authorization (PUMA). It covers the indication(s) and appropriate formulation(s) for medicines developed exclusively for use in the pediatric population, and, once obtained, it grants holders the benefit of 8 plus 2 years of data and market protection, provided that studies are performed as per an agreed (voluntary) PIP (6). The limited attractiveness of repurposing off-patent medicines through the PUMA scheme has been highlighted (Ruggieri et al., Wang and Giannuzzi); developers likely prefer to apply under the simplified procedure outlined in the Directive 2001/83/EC for off-patent drugs, rather than following the Pediatric Regulation route (Ruggieri et al.).

In the context of 20 projects funded under the Seventh Framework Programme for Research by the European Commission, three new PUMAs were obtained through the centralized procedure, and three of the 18 agreed PIPs were successfully completed within 3–10 years. Other outcomes were achieved, including the successful completion of pediatric clinical trials, development of new patents and technologies, dissemination of results through scientific publications, updates to clinical guidelines, EU-wide surveys on pediatric clinical practice, and advancement of the state-of-the-art in niche therapeutic subsets, such as neonatal-only diseases and pediatric pain.

Therefore, in addition to regulatory barriers and financial constraints, it seems that turning off-label uses into authorized ones is contingent upon the commercial interest.

## Formulations to comply with the specific patient needs

The lack of appropriate pediatric formulations is still a challenge for providing optimal treatment in children. Therefore, repurposing, including reformulation strategies, has the potential to address pediatric unmet needs and to ensure relevant “on-label” use. Wang and Giannuzzi discuss potential solutions to incentivise the development of new formulations for children, such as the *specific* inclusion of reformulation in repurposing R&D initiatives, *specific* R&D funding initiatives for reformulation, and *specific* measures and incentives at the regulatory level to attract manufacturers.

## Successes and failures

All these scenarios could potentially address unmet medical needs and enable relevant “on-label” clinical use, which are core elements of repurposing (Ambrosini et al., Fuchs et al., Ruggieri et al.).

Regardless of whether the results of a repurposing programme are positive or negative, or whether its promoter is academic or commercial, this Research Topic demonstrates that even unsuccessful programs can provide important insights into disease mechanisms, pave the way for new treatments and new studies, and generate innovation and attract interest from industry partners—provided they are based on a sound rationale, the study design is robust enough to ensure meaningful results, and the investigators keep the complete clinical development picture in mind (Ambrosini et al., Fuchs et al., Jonker et al., Ruggieri et al.).

“*No drug is ever understood completely, and repositioning, no matter how unlikely, often remains a possibility*” (7).

## Real world data and innovative methods providing evidence to endorse drug use

The use of real world data (RWD), pharmacometrics, artificial intelligence (AI), and other innovative methods has emerged due to the recognized benefits of generating evidence for the development of medicines for small populations and for supporting regulatory approvals when only limited data are available. As an example, the challenge for addressing an ultra-rare disease remains in a sufficient level of accessible knowledge on the drug and on the disease in order to derisk the development in the new indication (Paquot et al.). This Research Topic presents concrete applications of innovative methods in drug repurposing for small populations.

Healy et al. and Lava et al. showed that *model-based approaches* are useful in special pediatric settings characterized by unsatisfactory therapies for children, such as heart failure, or by practical and ethical challenges, such as those encountered in pediatric intensive care units. These approaches maximize information content and reduce the patient burden. The application of modeling, simulation, and extrapolation principles do inform about suitable dosing regimens, as demonstrated for lorazepam—currently used off-label for analgosedation in pediatric intensive care units, with limited data on its pharmacokinetics and pharmacodynamics (Healy et al.)—and in the context of a prospective trial with gliflozines, where a model-based approach can optimize study design, thereby increasing the probability of trial success (Lava et al.).

On the other hand, RWD can be analyzed to identify potential associations between existing drugs and new therapeutic indications, as well as to support post-marketing surveillance of both the safety and efficacy of approved products (Jonker et al.). Given the importance of quality and data curation for the use of RWD, the c4c consortium has worked on the standardization of pediatric clinical trial data, which can potentially be used as real world evidence (RWE) (Owen et al.).

Advanced computational methods, machine learning, AI, and platforms created or under construction in the EU, such as REMEDI4ALL and REPO4EU, are increasingly used to identify potential drug repurposing candidates (Jonker et al., Cortial et al.).

Systematic phenotypic screening (Paquot et al.), AI-driven initiatives, organoids, and gene expression datasets (Cortial et al.) catalyse the development of drugs for ultra-rare diseases by evaluating drug candidates based on their observable effects on cellular or organismal phenotypes. In addition, recent advancements in screening and computational techniques offer the unique opportunity to predict drug–disease interactions and prioritize candidate compounds for further investigation (Fuchs et al.). All of these tools and methods are expected to be useful if/when the proposed rules from the upcoming EU pharmaceutical legislation are implemented, including: (1) the possibility of including data on repurposing use in marketing authorization documents, and (2) the ability to conduct pediatric studies within a PIP for a disease different from the one proposed by the developer, based on the drug's mechanism of action.

## Conclusion

Taken together, the manuscripts included in the Research Topic “*Repurposing for small population needs*” provide a snapshot of the opportunities offered by drug repurposing for small populations.

Repurposing studies involving older products have the potential to generate innovation and attract interest from industry partners, provided they are based on a sound rationale, the study design is robust enough to ensure meaningful results, and the investigators keep the complete clinical development picture in mind (Ambrosini et al.). Importantly, this represents an opportunity not only to generate evidence but also to share clinical information and advance scientific knowledge.

In line with previous findings [*(**8**)* *Pinsent Masons;* *(**9*, *10**)*], the Research Topic “*Repurposing for small population needs*” also highlights the challenges and barriers encountered by academic and not-for-profit organizations (Ambrosini et al.; Ruggieri et al.):

- regulatory issues;
- limited financial resources and public funding, especially in a multi-national setting;
- low economic interest (cheaper options such as off-label medicines and extemporaneous galenic preparations are anyway available);
- complex negotiation of existing product intellectual property (IP), patents, and licensing agreements between non-commercial academic entities and companies, affecting the supply of both old and new drugs in the context of clinical studies;
- recruitment challenges in clinical trials due to limited feasibility, particularly in the context of unlicensed or off-label access.

Several initiatives have been launched in Europe to support drug repurposing, including innovation platforms, regulatory initiatives, funded projects and programmes. The outcomes of these emerging activities are highly anticipated for the benefit of patients, public health, and medicine developers.
